# 3-Hydr­oxy-*N*′-(5-hydr­oxy-2-nitro­benzyl­idene)-2-naphthohydrazide

**DOI:** 10.1107/S1600536809045279

**Published:** 2009-11-04

**Authors:** De-Suo Yang

**Affiliations:** a Department of Chemistry and Chemical Engineering, Baoji University of Arts and Sciences, Baoji 721007, People’s Republic of China

## Abstract

The mol­ecule of the title compound, C_18_H_13_N_3_O_5_, displays an *E* configuration with respect to the C=N double bond. The dihedral angle between the benzene ring and the naphthyl system is 1.1 (2)°. In the crystal structure, mol­ecules are linked through inter­molecular N—H⋯O and O—H⋯O hydrogen bonds, forming a three-dimensional network.

## Related literature

For the biological and structural chemistry of hydrazone compounds, see: Avaji *et al.* (2009 ▶); Charkoudian *et al.* (2007 ▶); Cukurovali *et al.* (2006 ▶). For related structures, see: Yang (2008*a* ▶,*b* ▶,*c* ▶,*d* ▶,*e*, 2007*a* ▶,*b* ▶,*c* ▶); Yang & Guo (2006 ▶).
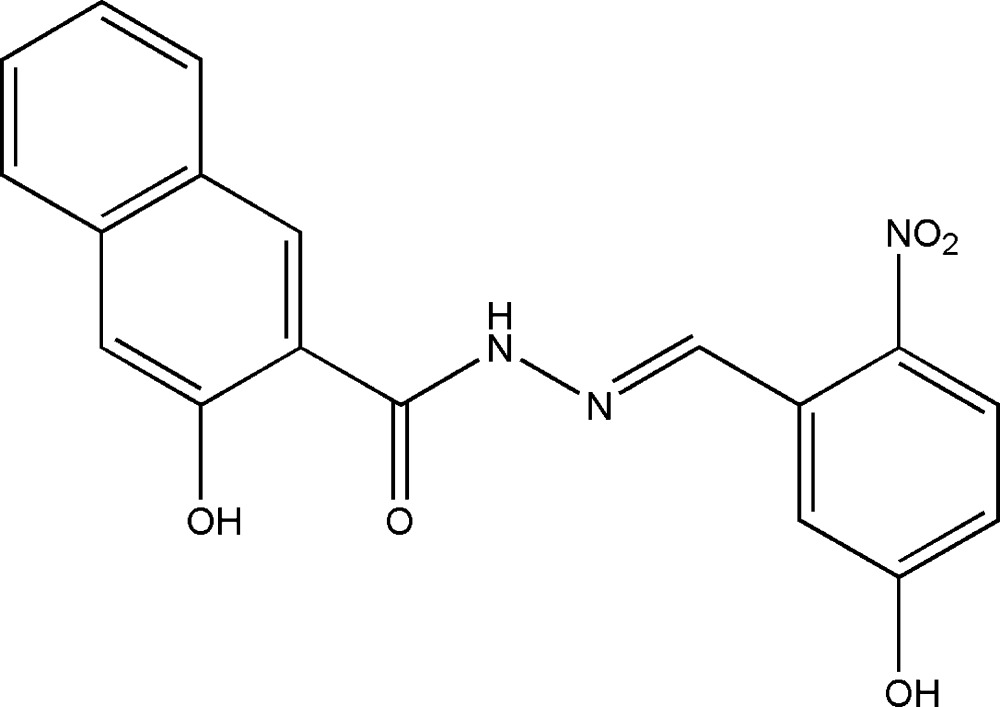


## Experimental

### 

#### Crystal data

C_18_H_13_N_3_O_5_
*M* *_r_* = 351.31
Monoclinic, 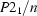

*a* = 10.1588 (3) Å
*b* = 8.2562 (2) Å
*c* = 19.5268 (5) Å
β = 104.867 (1)°
*V* = 1582.95 (7) Å^3^
*Z* = 4
Mo *K*α radiation
μ = 0.11 mm^−1^
*T* = 298 K
0.23 × 0.20 × 0.20 mm

#### Data collection

Bruker SMART CCD diffractometer
Absorption correction: multi-scan (*SADABS*; Sheldrick, 1996 ▶) *T*_min_ = 0.975, *T*_max_ = 0.978
9168 measured reflections
3425 independent reflections
2436 reflections with *I* > 2σ(*I*)
*R*_int_ = 0.028

#### Refinement

*R*[*F* ^2^ > 2σ(*F* ^2^)] = 0.044
*wR*(*F* ^2^) = 0.119
*S* = 1.04
3425 reflections
241 parameters
1 restraint
H atoms treated by a mixture of independent and constrained refinement
Δρ_max_ = 0.19 e Å^−3^
Δρ_min_ = −0.19 e Å^−3^

### 

Data collection: *SMART* (Bruker, 2002 ▶); cell refinement: *SAINT* (Bruker, 2002 ▶); data reduction: *SAINT*; program(s) used to solve structure: *SHELXS97* (Sheldrick, 2008 ▶); program(s) used to refine structure: *SHELXL97* (Sheldrick, 2008 ▶); molecular graphics: *SHELXTL* (Sheldrick, 2008 ▶); software used to prepare material for publication: *SHELXTL*.

## Supplementary Material

Crystal structure: contains datablocks global, I. DOI: 10.1107/S1600536809045279/bh2256sup1.cif

Structure factors: contains datablocks I. DOI: 10.1107/S1600536809045279/bh2256Isup2.hkl

Additional supplementary materials:  crystallographic information; 3D view; checkCIF report

## Figures and Tables

**Table 1:** Hydrogen-bond geometry (Å, °)

*D*—H⋯*A*	*D*—H	H⋯*A*	*D*⋯*A*	*D*—H⋯*A*
O2—H2⋯O1	0.82	1.88	2.5987 (18)	146
N1—H1⋯O4^i^	0.898 (9)	2.144 (10)	3.0361 (19)	172.8 (18)
O5—H5⋯O1^ii^	0.82	1.88	2.6889 (17)	168

Symmetry codes: (i) 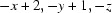; (ii) 
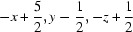
.

**Fig. 1.**
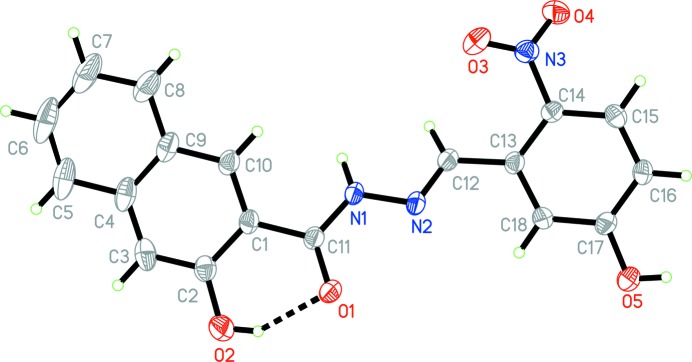
The structure of the title compound showing the atom-numbering scheme. Displacement ellipsoids are drawn at the 30% probability level. Hydrogen bond is shown as a dashed line.

**Fig. 2.**
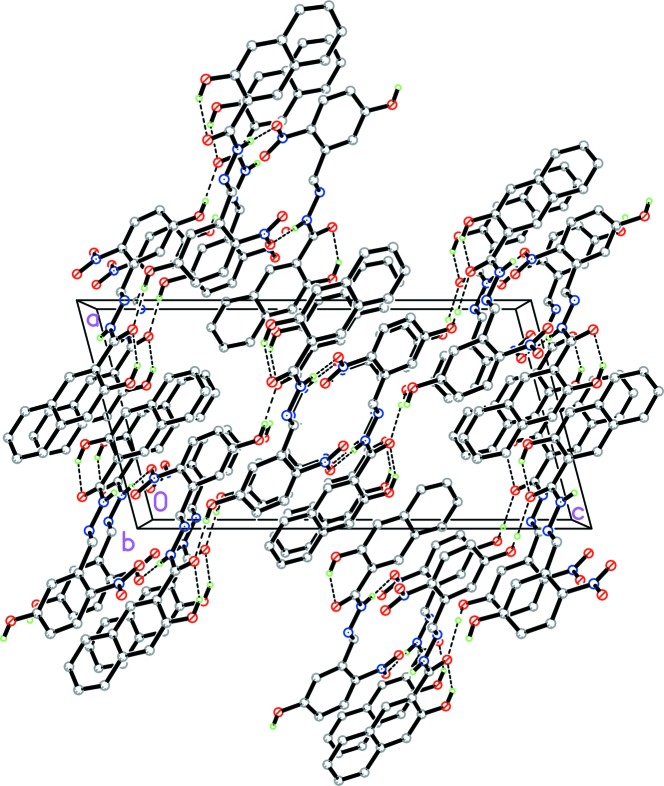
Molecular packing as viewed along the b axis. Hydrogen bonds are shown as dashed lines.

## supplementary crystallographic information

### Comment

Hydrazone compounds have been of great interest for their versatile biological and structural chemistry (Avaji *et al.*, 2009; Charkoudian *et al.*, 2007; Cukurovali *et al.*, 2006). Recently, we have reported a few hydrazone compounds (Yang, 2008*a*,*b*,*c*,*d*,*e*, 2007*a*,*b*,*c*; Yang & Guo, 2006). As a further investigation of this work, the crystal structure of the title new hydrazone compound is reported.

In the title compound (Fig. 1), the molecule displays an *E* configuration with respect to the C═N double bond. The C13···C18 benzene ring forms dihedral angles of 28.6 (2) and 1.1 (2)°, respectively, with the O3—N3—O4 nitro group and the C1···C10 naphthyl ring. All bond lengths are within normal ranges. The C12═N2 bond length of 1.266 (2) Å, conforms to the value for a formal double bond. The bond length of 1.341 (2) Å between atoms C11 and N1 is intermediate between a C—N single bond and a C═N double bond, because of conjugation effects in the molecule.

In the crystal structure, molecules are linked through intermolecular N—H···O and O—H···O hydrogen bonds (Table 1), forming a three-dimensional network (Fig. 2).

### Experimental

5-Hydroxy-2-nitrobenzaldehyde (0.1 mmol, 16.7 mg) and 3-hydroxy-2-naphthohydrazide (0.1 mmol, 20.2 mg) were dissolved in CHCl_3_ (10 ml). The mixture was stirred at room temperature to give a clear colorless solution. Crystals of the title compound were formed by gradual evaporation of the solvent over a period of 3 days, at room temperature.

### Refinement

Atom H1 was located in a difference map and refined isotropically, with N—H distance restrained to 0.90 (1) Å. Other H atoms were placed in idealized positions and constrained to ride on their parent atoms, with O—H distances of 0.82 Å, C—H distances of 0.93 Å, and with *U*_iso_(H) = 1.2*U*_eq_(C) and 1.5*U*_eq_(O).

### Crystal data

**Table:**

C_18_H_13_N_3_O_5_	*F*(000) = 728
*M**_r_* = 351.31	*D*_x_ = 1.474 Mg m^−^^3^
Monoclinic, *P*2_1_/*n*	Mo *K*α radiation, λ = 0.71073 Å
Hall symbol: -P 2yn	Cell parameters from 2463 reflections
*a* = 10.1588 (3) Å	θ = 2.5–26.6°
*b* = 8.2562 (2) Å	µ = 0.11 mm^−^^1^
*c* = 19.5268 (5) Å	*T* = 298 K
β = 104.867 (1)°	Block, colorless
*V* = 1582.95 (7) Å^3^	0.23 × 0.20 × 0.20 mm
*Z* = 4	

### Data collection

**Table:**

Bruker SMART CCD diffractometer	3425 independent reflections
Radiation source: fine-focus sealed tube	2436 reflections with *I* > 2σ(*I*)
graphite	*R*_int_ = 0.028
ω scans	θ_max_ = 27.0°, θ_min_ = 2.1°
Absorption correction: multi-scan (*SADABS*; Sheldrick, 1996)	*h* = −12→12
*T*_min_ = 0.975, *T*_max_ = 0.978	*k* = −10→10
9168 measured reflections	*l* = −24→16

### Refinement

**Table:**

Refinement on *F*^2^	Primary atom site location: structure-invariant direct methods
Least-squares matrix: full	Secondary atom site location: difference Fourier map
*R*[*F*^2^ > 2σ(*F*^2^)] = 0.044	Hydrogen site location: inferred from neighbouring sites
*wR*(*F*^2^) = 0.119	H atoms treated by a mixture of independent and constrained refinement
*S* = 1.04	*w* = 1/[σ^2^(*F*_o_^2^) + (0.0503*P*)^2^ + 0.3232*P*] where *P* = (*F*_o_^2^ + 2*F*_c_^2^)/3
3425 reflections	(Δ/σ)_max_ < 0.001
241 parameters	Δρ_max_ = 0.19 e Å^−^^3^
1 restraint	Δρ_min_ = −0.19 e Å^−^^3^
0 constraints	

### Fractional atomic coordinates and isotropic or equivalent isotropic displacement parameters (Å^2^)

**Table:**

	*x*	*y*	*z*	*U*_iso_*/*U*_eq_	
N1	0.88109 (14)	0.92607 (17)	0.05530 (7)	0.0462 (4)	
N2	1.00455 (13)	0.88709 (16)	0.10140 (7)	0.0446 (3)	
N3	1.20940 (14)	0.48961 (18)	0.04801 (7)	0.0461 (3)	
O1	0.86564 (13)	1.16125 (15)	0.11112 (6)	0.0575 (4)	
O2	0.63397 (15)	1.31345 (18)	0.08033 (7)	0.0689 (4)	
H2	0.7084	1.2854	0.1052	0.103*	
O3	1.13711 (14)	0.56430 (16)	−0.00173 (6)	0.0612 (4)	
O4	1.24674 (14)	0.34954 (16)	0.04217 (7)	0.0695 (4)	
O5	1.38574 (15)	0.75885 (19)	0.31532 (6)	0.0721 (4)	
H5	1.4565	0.7160	0.3377	0.108*	
C1	0.69456 (16)	1.10569 (19)	0.00579 (8)	0.0407 (4)	
C2	0.60609 (17)	1.2299 (2)	0.01840 (9)	0.0485 (4)	
C3	0.49048 (18)	1.2674 (3)	−0.03235 (10)	0.0601 (5)	
H3	0.4332	1.3482	−0.0234	0.072*	
C4	0.45611 (17)	1.1865 (3)	−0.09781 (10)	0.0582 (5)	
C5	0.3359 (2)	1.2237 (3)	−0.15133 (13)	0.0833 (8)	
H5A	0.2756	1.3013	−0.1428	0.100*	
C6	0.3090 (3)	1.1465 (4)	−0.21477 (15)	0.1025 (11)	
H6	0.2299	1.1717	−0.2493	0.123*	
C7	0.3980 (3)	1.0294 (3)	−0.22928 (13)	0.1001 (10)	
H7	0.3782	0.9787	−0.2733	0.120*	
C8	0.5134 (2)	0.9896 (3)	−0.17908 (11)	0.0769 (7)	
H8	0.5721	0.9116	−0.1889	0.092*	
C9	0.54456 (19)	1.0664 (2)	−0.11211 (9)	0.0541 (5)	
C10	0.66309 (18)	1.0299 (2)	−0.05915 (9)	0.0477 (4)	
H10	0.7224	0.9519	−0.0683	0.057*	
C11	0.81986 (16)	1.06728 (19)	0.06131 (8)	0.0417 (4)	
C12	1.04643 (17)	0.7455 (2)	0.09371 (9)	0.0468 (4)	
H12	0.9935	0.6774	0.0596	0.056*	
C13	1.17752 (16)	0.68736 (18)	0.13789 (8)	0.0398 (4)	
C14	1.25482 (16)	0.56598 (18)	0.11681 (8)	0.0381 (3)	
C15	1.37767 (16)	0.51385 (19)	0.16009 (9)	0.0429 (4)	
H15	1.4276	0.4342	0.1443	0.051*	
C16	1.42572 (16)	0.5796 (2)	0.22626 (8)	0.0440 (4)	
H16	1.5094	0.5471	0.2550	0.053*	
C17	1.34847 (18)	0.6951 (2)	0.24990 (8)	0.0466 (4)	
C18	1.22688 (17)	0.7487 (2)	0.20549 (8)	0.0481 (4)	
H18	1.1772	0.8280	0.2216	0.058*	
H1	0.8395 (18)	0.8513 (19)	0.0237 (8)	0.066 (6)*	

### Atomic displacement parameters (Å^2^)

**Table:**

	*U*^11^	*U*^22^	*U*^33^	*U*^12^	*U*^13^	*U*^23^
N1	0.0433 (8)	0.0423 (8)	0.0426 (8)	0.0066 (6)	−0.0077 (6)	−0.0034 (6)
N2	0.0416 (7)	0.0449 (8)	0.0399 (7)	0.0064 (6)	−0.0033 (6)	0.0013 (6)
N3	0.0441 (8)	0.0456 (8)	0.0486 (8)	−0.0067 (6)	0.0117 (7)	−0.0072 (7)
O1	0.0632 (8)	0.0469 (7)	0.0482 (7)	0.0076 (6)	−0.0119 (6)	−0.0087 (6)
O2	0.0754 (10)	0.0743 (10)	0.0563 (8)	0.0261 (8)	0.0159 (7)	−0.0026 (7)
O3	0.0687 (9)	0.0642 (9)	0.0433 (7)	−0.0101 (7)	0.0007 (6)	−0.0002 (6)
O4	0.0683 (9)	0.0559 (8)	0.0786 (9)	0.0076 (7)	0.0085 (7)	−0.0274 (7)
O5	0.0725 (10)	0.0899 (11)	0.0412 (7)	0.0308 (8)	−0.0088 (6)	−0.0124 (7)
C1	0.0390 (8)	0.0370 (8)	0.0416 (8)	−0.0019 (7)	0.0022 (7)	0.0075 (7)
C2	0.0475 (10)	0.0517 (10)	0.0469 (10)	0.0047 (8)	0.0133 (8)	0.0101 (8)
C3	0.0431 (10)	0.0710 (13)	0.0687 (13)	0.0150 (9)	0.0189 (9)	0.0243 (11)
C4	0.0378 (9)	0.0711 (13)	0.0591 (12)	−0.0109 (9)	0.0004 (8)	0.0282 (10)
C5	0.0427 (11)	0.1106 (19)	0.0836 (16)	−0.0086 (12)	−0.0074 (11)	0.0461 (15)
C6	0.0652 (15)	0.120 (2)	0.0910 (19)	−0.0332 (16)	−0.0366 (14)	0.0452 (18)
C7	0.113 (2)	0.0834 (18)	0.0686 (15)	−0.0412 (17)	−0.0400 (15)	0.0161 (13)
C8	0.0945 (16)	0.0613 (13)	0.0542 (12)	−0.0199 (12)	−0.0187 (11)	0.0049 (10)
C9	0.0536 (11)	0.0486 (10)	0.0485 (10)	−0.0154 (9)	−0.0080 (8)	0.0144 (8)
C10	0.0518 (10)	0.0375 (8)	0.0459 (9)	−0.0020 (8)	−0.0022 (8)	0.0045 (7)
C11	0.0437 (9)	0.0382 (8)	0.0384 (8)	0.0000 (7)	0.0016 (7)	0.0026 (7)
C12	0.0455 (9)	0.0440 (9)	0.0434 (9)	0.0023 (8)	−0.0022 (7)	−0.0052 (7)
C13	0.0403 (8)	0.0366 (8)	0.0387 (8)	0.0020 (7)	0.0031 (7)	0.0030 (7)
C14	0.0422 (8)	0.0333 (8)	0.0376 (8)	−0.0044 (7)	0.0082 (7)	0.0001 (6)
C15	0.0438 (9)	0.0363 (8)	0.0497 (9)	0.0043 (7)	0.0141 (8)	0.0035 (7)
C16	0.0409 (9)	0.0443 (9)	0.0430 (9)	0.0060 (7)	0.0038 (7)	0.0090 (7)
C17	0.0506 (10)	0.0494 (10)	0.0350 (8)	0.0053 (8)	0.0022 (7)	0.0009 (7)
C18	0.0499 (10)	0.0462 (9)	0.0436 (9)	0.0140 (8)	0.0037 (8)	−0.0029 (7)

### Geometric parameters (Å, °)

**Table:**

N1—C11	1.341 (2)	C5—H5A	0.9300
N1—N2	1.3812 (18)	C6—C7	1.402 (4)
N1—H1	0.898 (9)	C6—H6	0.9300
N2—C12	1.266 (2)	C7—C8	1.362 (3)
N3—O3	1.2235 (18)	C7—H7	0.9300
N3—O4	1.2315 (18)	C8—C9	1.414 (3)
N3—C14	1.448 (2)	C8—H8	0.9300
O1—C11	1.2378 (18)	C9—C10	1.404 (2)
O2—C2	1.357 (2)	C10—H10	0.9300
O2—H2	0.8200	C12—C13	1.469 (2)
O5—C17	1.343 (2)	C12—H12	0.9300
O5—H5	0.8200	C13—C18	1.382 (2)
C1—C10	1.376 (2)	C13—C14	1.399 (2)
C1—C2	1.426 (2)	C14—C15	1.384 (2)
C1—C11	1.479 (2)	C15—C16	1.371 (2)
C2—C3	1.364 (2)	C15—H15	0.9300
C3—C4	1.405 (3)	C16—C17	1.387 (2)
C3—H3	0.9300	C16—H16	0.9300
C4—C9	1.414 (3)	C17—C18	1.387 (2)
C4—C5	1.422 (3)	C18—H18	0.9300
C5—C6	1.357 (4)		
			
C11—N1—N2	120.64 (13)	C9—C8—H8	119.8
C11—N1—H1	120.7 (13)	C10—C9—C4	118.26 (17)
N2—N1—H1	118.4 (13)	C10—C9—C8	122.1 (2)
C12—N2—N1	114.48 (14)	C4—C9—C8	119.63 (18)
O3—N3—O4	122.43 (14)	C1—C10—C9	122.28 (17)
O3—N3—C14	120.03 (14)	C1—C10—H10	118.9
O4—N3—C14	117.54 (14)	C9—C10—H10	118.9
C2—O2—H2	109.5	O1—C11—N1	121.64 (14)
C17—O5—H5	109.5	O1—C11—C1	121.38 (15)
C10—C1—C2	118.58 (15)	N1—C11—C1	116.98 (14)
C10—C1—C11	122.05 (15)	N2—C12—C13	120.90 (15)
C2—C1—C11	119.31 (14)	N2—C12—H12	119.5
O2—C2—C3	118.43 (17)	C13—C12—H12	119.5
O2—C2—C1	121.50 (15)	C18—C13—C14	116.75 (14)
C3—C2—C1	120.07 (17)	C18—C13—C12	119.55 (15)
C2—C3—C4	121.34 (18)	C14—C13—C12	123.64 (14)
C2—C3—H3	119.3	C15—C14—C13	121.96 (14)
C4—C3—H3	119.3	C15—C14—N3	116.99 (14)
C3—C4—C9	119.42 (16)	C13—C14—N3	121.04 (14)
C3—C4—C5	122.1 (2)	C16—C15—C14	119.98 (15)
C9—C4—C5	118.4 (2)	C16—C15—H15	120.0
C6—C5—C4	120.2 (3)	C14—C15—H15	120.0
C6—C5—H5A	119.9	C15—C16—C17	119.39 (15)
C4—C5—H5A	119.9	C15—C16—H16	120.3
C5—C6—C7	121.3 (2)	C17—C16—H16	120.3
C5—C6—H6	119.4	O5—C17—C16	122.72 (15)
C7—C6—H6	119.4	O5—C17—C18	117.25 (16)
C8—C7—C6	120.1 (3)	C16—C17—C18	120.03 (15)
C8—C7—H7	119.9	C13—C18—C17	121.79 (16)
C6—C7—H7	119.9	C13—C18—H18	119.1
C7—C8—C9	120.3 (3)	C17—C18—H18	119.1
C7—C8—H8	119.8		

### Hydrogen-bond geometry (Å, °)

**Table:**

*D*—H···*A*	*D*—H	H···*A*	*D*···*A*	*D*—H···*A*
O2—H2···O1	0.82	1.88	2.5987 (18)	146
N1—H1···O4^i^	0.90 (1)	2.14 (1)	3.0361 (19)	173 (2)
O5—H5···O1^ii^	0.82	1.88	2.6889 (17)	168

Symmetry codes: (i) −*x*+2, −*y*+1, −*z*; (ii) −*x*+5/2, *y*−1/2, −*z*+1/2.
